# ZDHHC介导的蛋白质棕榈酰化在NSCLC发生发展及免疫逃逸中的研究进展及其应用

**DOI:** 10.3779/j.issn.1009-3419.2025.102.15

**Published:** 2025-04-20

**Authors:** Wangcheng CHEN, Lili PANG, Yuemei LAN, Yanhong SHI, Bingbing WEN, Baihong ZHANG

**Affiliations:** ^1^730050 兰州，西北民族大学医学部（陈王成，史彦红，文兵兵）; ^1^Northwest Minzu University College of Medicine; ^2^中国人民解放军联勤保障部队第940医院肿瘤科（陈王成，庞丽丽，兰月梅，张百红）; ^2^Department of Oncology, the 940^th^ Hospital of Joint Logistic Support Force of Chinese People's Liberation Army; ^3^甘肃中医药大学第一临床学院（庞丽丽，兰月梅）; ^3^Gansu University of Chinese Medicine First School of Clinical Medical, Lanzhou 730050, China

**Keywords:** 肺肿瘤, ZDHHC酶, 蛋白质棕榈酰化, 免疫逃逸, PD-L1, Lung neoplasms, ZDHHC enzymes, Protein palmitoylation, Immune escape, PD-L1

## Abstract

非小细胞肺癌（non-small cell lung cancer, NSCLC）作为全球癌症死亡的主要原因，尽管免疫检查点抑制剂的治疗取得进展，但耐药性仍是一个重大的临床挑战。棕榈酰化是一种关键的蛋白质翻译后修饰（post- translational modifications, PTM），主要由含锌指DHHC结构型（zinc finger DHHC-type, ZDHHC）的棕榈酰基转移酶催化，近年研究表明其在NSCLC中具有重要意义。本综述旨在阐明ZDHHC介导的蛋白质棕榈酰化在NSCLC发生发展和免疫逃逸中的作用机制及临床应用前景。

## Abstract (en)

Non-small cell lung cancer (NSCLC), a leading cause of cancer-related deaths worldwide, remains a significant clinical challenge despite advances in immune checkpoint inhibitors therapy, with drug resistance persisting as a major obstacle. Palmitoylation, a critical post-translational modification (PTM) primarily catalyzed by palmitoyltransferases of the zinc finger DHHC-type (ZDHHC), has recently demonstrated important implications in NSCLC. This review aims to elucidate the mechanisms and clinical potential of ZDHHC-mediated protein palmitoylation in NSCLC progression and immune escape.

非小细胞肺癌（non-small cell lung cancer, NSCLC）占肺癌病例的80%-85%，是全球癌症相关死亡的主要原因^[1]^。尽管免疫治疗，特别是针对程序性细胞死亡受体（programmed cell death 1, PD-1）和程序性细胞死亡配体1（programmed cell death ligand 1, PD-L1）轴的免疫检查点抑制剂（immune checkpoint inhibitors, ICIs）取得了进展，但仍有相当一部分患者由于肿瘤免疫逃逸机制而表现出原发性或获得性耐药^[2]^。有研究^[3]^表明，蛋白质棕榈酰化修饰是恶性肿瘤中致癌信号传导和免疫调节的关键调节形式。

蛋白质棕榈酰化是一种可逆的脂质修饰，由含锌指DHHC结构域（zinc finger DHHC-type, ZDHHC）的棕榈酰基转移酶催化，它能调节底物蛋白的膜定位、稳定性和相互作用^[4]^。目前已鉴定出超过23种ZDHHC异构体，其中特定成员通过异常激活表皮生长因子受体（epidermal growth factor receptor, EGFR）、RAS和Wnt/β-catenin等通路参与肿瘤进展^[5-7]^。Zhang等^[8]^研究表明，棕榈酰化通过调节PD-L1表达和改变肿瘤-免疫细胞相互作用参与免疫逃逸。研究^[9,10]^发现ZDHHC9通过棕榈酰化调节PD-L1稳定性，从而促进肺腺癌的发生与发展。相反，ZDHHC9介导的脂肪酸合成酶棕榈酰化能促进脂筏形成，增强了免疫抑制性细胞因子的分泌。

尽管取得了这些进展，对于ZDHHC酶如何协调NSCLC免疫逃逸的系统性理解仍然缺乏。现有文献缺少将机制洞察与治疗发展相结合的整合，特别是在克服ICIs耐药的组合策略背景下的整合。本综述综合了关于ZDHHC驱动的棕榈酰化在NSCLC免疫逃逸中的最新发现，评估了临床前治疗方法，并展望了未来研究的方向。通过阐明ZDHHC在肿瘤发生和免疫调节中的双重作用，本文旨在整合转化差距，并激发新的治疗模式。

## 1 蛋白质棕榈酰化与ZDHHC酶的基本机制

蛋白质棕榈酰化是一种动态且可逆的翻译后修饰（post-translational modification, PTM），通过硫酯键将棕榈酸共价连接到目标蛋白质的半胱氨酸残基上，在蛋白质运输、膜锚定以及细胞信号网络内的相互作用中发挥关键调控作用^[11]^。与豆蔻酰化或异戊烯化等其他脂质修饰不同，棕榈酰化的可逆性使其能够对蛋白质的定位和活性进行精细的时空调控，从而成为细胞生理学中的多功能调节形式^[12]^。这一过程的核心是ZDHHC酶，它是一类膜结合酰基转移酶，负责催化棕榈酸从棕榈酰辅酶A转移到底物蛋白质上。棕榈酰化主要分为两种形式：S-棕榈酰化（硫酯键连接）和较少见的N-棕榈酰化（酰胺键连接），其中S-棕榈酰化是哺乳动物中的主要形式，且完全由ZDHHC酶介导^[13]^。这些酶具有一个保守的催化基序Asp-His-His-Cys，该基序嵌入在一个富含半胱氨酸的结构域中，催化了两步反应机制：首先，ZDHHC酶在其活性位点的半胱氨酸上发生自棕榈酰化，形成棕榈酰-酶中间体；随后，棕榈酰基被转移到底物半胱氨酸的硫醇基上^[14]^。这种修饰的可逆性由酰基蛋白硫酯酶（acyl protein thioesterase, APT）和棕榈酰蛋白硫酯酶（palmitoyl protein thioesterase, PPT）调控，例如APT1/2和PPT1，它们通过水解硫酯键来去棕榈酰化蛋白质^[15]^。这种动态平衡确保了蛋白质功能的精确控制，例如通过快速的棕榈酰化和去棕榈酰化循环来调节信号蛋白如RAS和SRC激酶在质膜和细胞内区室之间的穿梭^[5]^。

人类基因组编码了23种ZDHHC异构体（ZDHHC1-24，其中ZDHHC10被注释为假基因），每种异构体均表现出独特的底物特异性、组织分布和亚细胞定位^[16,17]^。从结构上看，ZDHHC酶的特征包括1个对催化活性至关重要的ZDHHC-CRD基序，其中突变（如Cys到Ser的替换）会消除棕榈酰转移酶功能；这些酶通常含有4-6个跨膜结构域（transmembrane domains, TMDs），使其锚定于内质网、高尔基体或质膜等不同细胞器上，以及可变的N端和C端区域，介导底物识别、酶二聚化以及与调控蛋白的相互作用^[18]^。例如，ZDHHC5和ZDHHC20定位于质膜，主要催化跨膜蛋白PD-L1的棕榈酰化修饰；而ZDHHC2和ZDHHC3驻留于高尔基体，负责修饰分泌蛋白或囊泡相关蛋白^[8,19]^。

棕榈酰化通过调节3个关键方面影响着细胞生理学：膜结合、蛋白质稳定性和信号传导。首先，棕榈酰化增强了与脂质双层的疏水相互作用，通常与其他脂质修饰如SRC激酶中的肉豆蔻酰化协同作用，例如，RAS癌基因家族成员（member RAS oncogene family, RAB27B）通过募集ZDHHC9介导的脂肪酸合成酶棕榈酰化，促进质膜定位^[20]^。其次，它可以保护蛋白质免受泛素化和蛋白酶体降解，如ZDHHC5介导的EGFR在Cys^797^处的棕榈酰化，阻止了溶酶体分选并延长了NSCLC中的致癌信号^[21]^。第三，棕榈酰化通过将信号分子聚集到脂筏（富含胆固醇的膜微域）中，促进高效的信号传导。例如，ZDHHC5和ZDHHC8在背根节轴突中富集，调控Gp130/JAK/STAT3信号通路和DLK/JNK通路，从而在轴突损伤反应中发挥重要作用^[22]^。新出现的证据还强调了棕榈酰化与其他PTM之间的相互作用；例如，敲除酰基辅酶A合成酶长链家族成员5（acyl-CoA synthetase long chain family member 5, ACSL5）基因会影响Wnt3a的棕榈酰化修饰，从而抑制了Wnt/β-catenin通路的激活^[23]^。

## 2 ZDHHC酶在NSCLC发病机制中的作用

ZDHHC酶通过调控关键致癌蛋白的活性与定位，在NSCLC的发生、转移及治疗抵抗中发挥重要作用。研究表明，多个ZDHHC酶在NSCLC中异常高表达。例如，ZDHHC5在约40%的NSCLC病例中过表达^[24]^，ZDHHC5会影响PD-L1的膜定位，继而影响膜的定向，从而调节其与T细胞表面PD-1的结合和免疫调节^[8]^。其分子机制涉及对PD-L1的棕榈酰化修饰：棕榈酰化增强PD-L1对脂筏的亲和力，使其定位于有序膜区域，并通过抑制泛素化和溶酶体降解维持其稳定性。在有序膜区域，PD-L1倾向于采取“平卧”构象，减少与PD-1的空间接触，促进T细胞杀伤癌细胞；而棕榈酰化能改变PD-L1的膜分布和构象，动态调节其与PD-1的相互作用，影响肿瘤免疫逃逸^[8]^。Enrico等^[25]^研究表明，ZDHHC20-BRAF融合基因始终与原始EGFR激活突变共存。在4例伴有T790M突变的NSCLC患者中，3例在出现ZDHHC20-BRAF融合基因时丢失了T790M突变。ZDHHC20的上调可促进EGFR与HER2的异源二聚化，导致奥希替尼（Osimertinib）治疗耐药^[25,26]^。实验^[27]^证明，ZDHHC20通过对YTHDF3的Cys^474^位点进行S-棕榈酰化，抑制其伴侣介导的自噬降解，导致癌基因产物MYC异常积累，从而增强癌细胞恶性表型，靶向ZDHHC20-YTHDF3-MYC信号轴的棕榈酰化调控在癌症治疗方面具有潜力。

ZDHHC酶的治疗策略受到其功能复杂性的挑战，但其精准干预措施已展现出临床前景。例如，敲低ZDHHC20或表达棕榈酰化抗性EGFR突变体可阻断EGFR棕榈酰化，从而抑制磷脂酰肌醇3-激酶（phosphatidylinositol 3-kinase, PI3K）激活、降低MYC转录因子水平、减少细胞增殖，并在KRAS突变肺腺癌小鼠模型中显著抑制肿瘤生长。此外，ZDHHC20与EGFR-酪氨酸激酶抑制剂（EGFR-tyrosine kinase inhibitors, EGFR-TKIs）耐药相关，其抑制剂通过干扰突变EGFR的膜定位可有效逆转耐药^[28]^。在KRAS突变型NSCLC中，ZDHHC9介导的KRAS4A棕榈酰化不仅增强其致癌信号传导，还通过修饰葡萄糖转运蛋白GLUT1促进肿瘤糖酵解代谢^[13]^。此外，临床数据^[29]^提示ZDHHC表达谱可作为生物标志物：ZDHHC高表达的肿瘤对PD-1抑制剂反应较差，而联合ZDHHC抑制剂可显著提升疗效。上述发现不仅揭示了ZDHHC在NSCLC中的复杂调控机制，也为开发基于棕榈酰化修饰的精准疗法提供了理论依据。

## 3 ZDHHC酶在NSCLC中的免疫调节作用

ZDHHC酶通过棕榈酰化调控免疫相关蛋白的活性与稳定性，在NSCLC的免疫逃逸中发挥重要作用。研究^[30]^发现，ZDHHC酶可通过多种分子机制重塑肿瘤微环境（tumor microenvironment, TME），促进免疫抑制并削弱抗肿瘤免疫应答。

PD-L1是NSCLC免疫治疗的靶标，而ZDHHC酶对其功能的调控至关重要。多项研究^[31,32]^证实，ZDHHC9通过催化PD-L1的棕榈酰化修饰，增强其膜定位及稳定性。ZDHHC9抑制NSCLC细胞增殖、迁移和侵袭，同时刺激细胞凋亡，同时ZDHHC9敲除可以通过降低其棕榈酰化水平来促进PD-L1蛋白降解。PD-L1蛋白水平的降低可以增强抗肿瘤免疫力并抑制肺腺癌细胞的生长^[32]^。这直接导致PD-L1在肿瘤细胞表面蓄积，促进其与T细胞表面的PD-1结合，诱发T细胞耗竭。更重要的是，阻断PD-L1棕榈酰化可增强肿瘤细胞对T细胞杀伤的敏感性，从而抑制体内肿瘤生长^[30]^。与此同时，ZDHHC9在癌症中显著上调，被确认为一个癌基因。其表达受特异性蛋白1（specificity protein, SP1）激活调控，促进肿瘤发生与发展。Li等^[33]^研究表明ZDHHC9在膀胱癌中通过在Cys^420^位点棕榈酰化结合免疫球蛋白（binding immunoglobulin protein, Bip），抑制未折叠蛋白反应，从而提高Bip的蛋白质稳定性并维持其在内质网中的定位。此外，在肺腺癌中，ZDHHC9通过催化PD-L1的棕榈酰化修饰，增强PD-L1的膜定位和稳定性，促进其在细胞表面的积累。这使得PD-L1更有效地与T细胞表面的PD-1结合，诱发T细胞耗竭，进而促进肿瘤免疫抑制。

除PD-L1外，ZDHHC还调控T淋巴细胞免疫球蛋白黏蛋白3（T cell immunoglobulin and mucin domain-containing protein 3, TIM-3）等关键免疫检查点。例如，TIM-3在残基Cys^296^棕榈酰化，能阻止其与羟甲基戊二酸单酰辅酶A还原酶降解蛋白1（HMG-CoA reductase degradation protein 1, HRD1）结合而稳定TIM-3，从而抑制其多泛素化降解。ZDHHC9敲除减弱了嵌合抗原受体T细胞免疫疗法（chimeric antigen receptor T-cell immunotherapy, CAR-T）细胞耗竭，TIM-3棕榈酰化的肽抑制剂加速了TIM-3降解并增强了CAR-T细胞和自然杀伤细胞介导的抗肿瘤免疫^[34]^。ZDHHC5通过棕榈酰化内着丝粒蛋白（inner centromere protein, INCENP）的Cys^15^位点，可能影响其着丝粒定位功能，从而调控肺腺癌的干细胞特性，并与肿瘤侵袭性呈正相关^[24]^。动物实验^[35]^表明，ZDHHC5催化NLR家族含PYRIN域蛋白3（NLR family, pyrin domain-containing protein 3, NLRP3）的棕榈酰化，发生于其LRR结构域，通过促进NLRP3寡聚化以及与NIMA相关激酶7（NIMA-related kinase 7, NEK7）的相互作用，驱动炎症体组装，进而激活半胱氨酸的天冬氨酸蛋白水解酶1，释放白介素1β（interleukin 1β, IL-1β）和IL-18，并引发焦孔素D（gasdermin D, GSDMD）介导的细胞焦亡。此外，棕榈酰化的干扰素基因刺激因子（stimulator of interferon genes, STING）蛋白抑制干扰素通路激活，导致I型干扰素（interferon-I, IFN-I）分泌减少^[36]^。使用棕榈酰化抑制剂可恢复STING通路活性后，CD8^+^ T细胞浸润增加3.5倍，显著延缓肿瘤生长。

除上述免疫调控外，棕榈酰化还在肿瘤的发生和发展中有着重要作用。ZDHHC通过影响癌细胞的生长、分裂及侵袭能力，直接驱动肿瘤的病理演进。如ZDHHC9在膀胱癌中呈现高表达，其高表达与膀胱癌的分化程度和临床分期相关。同时，ZDHHC9还能通过棕榈酰化修饰EGFR和Bip蛋白，提升膀胱癌细胞的生存能力，并通过减弱未折叠蛋白反应维持内质网功能的稳定^[33]^。除此之外，ZDHHC5通过对INCENP的Cys^15^位点棕榈酰化调控着丝粒活动和染色体分配，ZDHHC5不同于其他的ZDHHC家族，其mRNA和蛋白水平上调，与恶性预后相关，敲除ZDHHC5可显著降低肺腺癌的肿瘤负荷和远处转移率，ZDHHC5与INCENP表达呈正相关，其相关性随肺腺癌分期增强，从而促进NSCLC的发生和侵袭性进展^[24]^。另一方面，棕榈酰化的异常活跃还与肿瘤的生物合成密切相关，ZDHHC3通过调控C^264^位点的SREBP与固醇调节元件结合蛋白裂解激活蛋白（SREBP cleavage-activating protein, SCAP）泛素化增强脂质合成，为肿瘤细胞快速增殖提供物质基础^[37]^。

针对上述机制，开发选择性ZDHHC抑制剂已成为突破免疫耐药性的新方向。例如，ZDHHC3通过2-溴棕榈酸抑制棕榈酰化或沉默ZDHHC3，可在体外及小鼠模型中激活抗肿瘤免疫，PD-L1在其细胞质域发生棕榈酰化，这一脂质修饰通过阻断泛素化稳定PD-L1，抑制其溶酶体降解^[29]^。此外，ZDHHC3在SCAP的C^264^位点进行棕榈酰化，拮抗人E3泛素蛋白连接酶（HECT domain and ankyrin repeat containing E3 ubiquitin protein ligase 1, HACE1）蛋白介导的SCAP泛素化，从而稳定SCAP，ZDHHC3小分子抑制剂联合抗PD-1免疫疗法在小鼠DEN/CCl4诱导的模型中抑制肿瘤生长^[37]^。Nagoya等^[38]^研究表明细胞骨架关联蛋白4（cytoskeleton-associated protein 4, CKAP4）通过外泌体分泌，受棕榈酰化调控，肺癌患者中CKAP4阳性表达与较差预后相关。CKAP4过表达促进体外细胞增殖和体内肿瘤生长，抗CKAP4抗体可抑制此效应。抗CKAP4抗体或奥希替尼单独抑制AKT活性、球体形成及异种移植肿瘤生长，二者联合效果更强。Kharbanda等^[28]^研究发现，抑制ZDHHC20可阻断EGFR棕榈酰化，显著削弱KRAS突变肺腺癌中MAPK与PI3K信号的二元切换，从而恢复EGFR抑制剂奥希替尼对T790M耐药突变细胞的敏感性。这一机制提示，ZDHHC20介导的EGFR棕榈酰化在KRAS驱动的信号饱和中起到关键作用，抑制ZDHHC20可减少PI3K复合物形成，降低肿瘤进展速度^[39]^。开发ZDHHC20抑制剂以降低EGFR-PI3K信号可能惠及KRAS突变肿瘤患者。

## 4 ZDHHC酶的免疫治疗

随着KRAS G12C抑制剂（索托拉西布/阿达格拉西布）、第三代EGFR-TKIs（奥希替尼）及间质上皮转化因子（mesenchymal to epithelial transition, MET）抑制剂（卡马替尼）等靶向药物的临床突破，研究者开始探索靶向蛋白质PTM的新方向，其中ZDHHC酶介导的棕榈酰化调控成为NSCLC免疫治疗的热点。研究^[3,30]^揭示，ZDHHC酶通过动态调控PD-L1、EGFR及免疫相关信号通路的棕榈酰化修饰，在免疫逃逸与治疗抵抗中发挥关键作用。周容等^[40]^通过基因表达综合数据库（Gene Expression Omnibus, GEO）和基因表达谱数据动态分析数据库（Gene Expression Profilling Interactive Analysis, GEPIA）分析发现，ZDHHC9在NSCLC组织中较癌旁组织显著升高，并且ZDHHC9表达水平与患者的无病生存率相关，这提示ZDHHC9可能是治疗相关的一个重要位点。在靶向治疗增效方面，实验证实ZDHHC9通过棕榈酰化调节PD-L1稳定性在NSCLC中的促瘤作用，突出了ZDHHC9作为NSCLC新的治疗靶点^[32]^，它还可通过阻断PD-1的Cys^192^位点棕榈酰化，促进其溶酶体降解，从而逆转PD-1单抗耐药^[41]^。此外，针对EGFR通路，RAB27A可在mRNA和蛋白质水平上调控ZDHHC13，影响其与EGFR的相互作用，从而改变EGFR的棕榈酰化水平，促进EGFR在癌细胞膜上的稳定表达^[42]^，因此，RAB27A是对抗侵袭性癌症的潜在治疗靶点。Kadry等^[26]^研究表明，在KRAS驱动的肺腺癌小鼠模型中通过CRISPR/Cas9技术抑制ZDHHC20基因能有效抑制肿瘤形成，这种抑制肿瘤效应是通过EGFR介导的，表达棕榈酰化抗性EGFR突变体同样能抑制KRAS驱动的肺腺癌发生，减少EGFR棕榈酰化能提高多种癌细胞对现有EGFR抑制剂和下游信号通路抑制剂的敏感性。生物标志物开发方面，基于质谱的PD-L1棕榈酰化定量检测（S-palmitoylation指数）已用于预测ZDHHC9抑制剂的敏感性，而ZDHHC17被确定为NLRP3抑制剂响应的标志物^[43]^。在先天免疫激活过程中，敲除ZDHHC1可通过抑制STING蛋白第88/91位半胱氨酸残基的棕榈酰化修饰^[44,45]^，显著增强STING通路活化，进而使TME中CD103^+ ^树突状细胞的浸润量提升3倍，并与细胞毒性T淋巴细胞相关抗原4（cytotoxic T lymphocyte-associated antigen 4, CTLA-4）抑制剂治疗产生空间协同效应。

## 5 总结与展望

棕榈酰化修饰通过影响PD-L1与TIM-3免疫检查点膜定位及稳定性，在NSCLC免疫逃逸中起到重要作用。前期的临床前研究^[30,32,46,47]^表明ZDHHC抑制剂能够改变肿瘤免疫微环境、增强抗PD-1治疗效果并克服耐药性，但仍存在一些转化难题，如ZDHHC亚型功能重叠、药物难以穿透血脑屏障和可能的非特异性毒性反应。今后研究应从三个方向着手：首先，利用ZDHHC酶结构特点结合人工智能技术，研发针对性更强的抑制剂；其次，研制外泌体递送系统，提高药物在脑转移灶的浓度；最后，尝试将棕榈酰化抑制与放疗和STING通路激活剂组合，调动多途径抗癌免疫反应。临床转化优先考虑PD-L1棕榈酰化水平高及ZDHHC亚型过表达的NSCLC患者亚群，同时建立相应生物标志物评估体系，最终达成"个体化棕榈酰化免疫治疗"目标。
